# Systemic corticosteroids in asthma: A call to action from World Allergy Organization and Respiratory Effectiveness Group^☆^

**DOI:** 10.1016/j.waojou.2022.100726

**Published:** 2022-12-10

**Authors:** Eugene R. Bleecker, Mona Al-Ahmad, Leif Bjermer, Marco Caminati, Giorgio Walter Canonica, Alan Kaplan, Nikolaos G. Papadopoulos, Nicolas Roche, Dermot Ryan, Yuji Tohda, Anahí Yáñez, David Price

**Affiliations:** a Division of Genetics, Genomics and Precision Medicine, Department of Medicine, University of Arizona, Tucson, AZ, USA; b Microbiology Department, College of Medicine, Kuwait University, Kuwait City, Kuwait; c Department of Respiratory Medicine and Allergology, University Hospital, Lund, Sweden; d Department of Medicine, University of Verona, Verona, Italy; e Department of Biomedical Sciences, Humanitas University, Pieve Emanuele, Italy; f Personalized Medicine, Asthma and Allergy, Humanitas Clinical and Research Center, IRCCS, Rozzano, Italy; g Family Physician Airways Group of Canada, Stouffville, ON, Canada; h Department of Family and Community Medicine, University of Toronto, Toronto, ON, Canada; i Division of Infection, Immunity & Respiratory Medicine, Royal Manchester Children's Hospital, University of Manchester, Manchester, UK; j Allergy Department, 2nd Pediatric Clinic, Athens General Children's Hospital ‘P&A Kyriakou’, University of Athens, Athens, Greece; k Department of Respiratory Medicine, APHP. Centre Université Paris Cité, Cochin Hospital (AP-HP) and Institute (UMR1016), Paris, France; l Usher Institute, University of Edinburgh, Edinburgh, UK; m Kindai University Hospital, Osaka, Japan; n Center for Research on Allergies and Respiratory Diseases (InAER), Buenos Aires, Argentina; o Observational and Pragmatic Research Institute, Singapore; p Centre of Academic Primary Care, Division of Applied Health Sciences, University of Aberdeen, Aberdeen, UK

**Keywords:** Severe asthma, Systemic corticosteroids, Adverse effects, Burden

## Abstract

Systemic corticosteroids (SCS) are a highly effective treatment for acute exacerbations and long-term symptom control in asthma. Long-term SCS use is highly prevalent across all asthma severities, occurring in over 20% of patients with severe or uncontrolled disease globally. It is now well known that exposure to both long-term and repeated acute courses of SCS is associated with a high risk of serious adverse effects (AEs), such as osteoporosis, and metabolic and cardiovascular complications, especially when prescribed onto a background of other corticosteroids. The aim of this call-to-action article, endorsed by the World Allergy Organization and the Respiratory Effectiveness Group, is to review the accumulating evidence on the burden of SCS on patients with asthma and provide an overview of potential strategies for implementing SCS Stewardship.

Primary prevention of exacerbations and improvement of asthma control is a key first step in achieving SCS Stewardship, by optimizing maintenance asthma medications and addressing modifiable risk factors, such as adherence and inhaler technique. Other key elements of SCS Stewardship include increasing appropriate specialist referrals for multidisciplinary review, assessment of biomarkers, and consideration of oral corticosteroid-sparing add-on therapies (eg, biologics). In cases where SCS use is deemed clinically justified, it should be tapered to the lowest possible dose. In addition, patients receiving long-term SCS or frequent acute courses should be closely monitored for emergence of SCS-related AEs.

Because of the extensive data available on the costly and burdensome AEs associated with SCS use, as well as the range of treatment options now available, there is a need for healthcare providers (HCPs) to carefully evaluate whether the benefits of SCS outweigh the potential harms, to adopt SCS-sparing and Stewardship strategies, and to consider alternative therapies where possible. Development of a structured and collaborative SCS Stewardship approach is urgently required to protect patients from the potential harm of SCS use.

## Introduction

Asthma is a heterogenous disease, characterized by chronic airway inflammation, that affects 1–18% of the population in different countries.^1^ Approximately 5–10% of the overall asthma population have severe asthma,^2^ defined as uncontrolled asthma despite adherence to maximal optimized inhaled corticosteroid (ICS)/long-acting β_2_-agonist (LABA) treatment and management of contributory factors.^1^ Acute courses of systemic corticosteroids (SCS; see Supplemental Appendix 1), encompassing both oral corticosteroids (OCS) and injectable corticosteroids, remain a key element in the treatment and management of severe asthma exacerbations.^1^ Despite increasing awareness of SCS-related adverse effects (AEs), globally long-term SCS therapy (see Supplemental Appendix 1) continues to be prescribed in patients with asthma experiencing uncontrolled symptoms and/or exacerbations despite optimized treatment.^3^

A wealth of data on the burdensome and costly AEs associated with SCS use in asthma now exists.^4^, ^5^, ^6^, ^7^ The data have informed updates to international/national recommendations on OCS use and prompted the publication of consensus statements/position papers highlighting the need to adopt OCS-sparing strategies.^8^, ^9^, ^10^, ^11^ There are now growing calls for a collaborative, systematic effort to implement SCS Stewardship in asthma management,^12^^,^^13^ similar to the successful programs that support Antimicrobial Stewardship.^9^ Effective SCS Stewardship has been achieved in other therapy areas, such as rheumatoid arthritis,^14^ and should be considered a realistic target in asthma. In an Australian database study on patients with rheumatoid arthritis (N = 3699), a reduction in corticosteroids (CS) was seen between 2001 and 2015,^14^ which was attributed to the increased awareness of CS-related AEs and improved availability of other disease-modifying therapies.^14^

This call to action, endorsed by the World Allergy Organization and the Respiratory Effectiveness Group, aims to highlight the prevalence and burden associated with inappropriate SCS use (SCS use that is not clinically justified; see also Supplemental Appendix 1) and provide a summary of potential strategies for implementing SCS Stewardship in asthma.^12^

### Prevalence and patterns of SCS use

Despite the introduction of updated treatment strategies and novel therapies for asthma, such as maintenance and reliever therapy and biologics, respectively, SCS use remains high. In a UK study examining longitudinal SCS use in patients across 28 conditions from 1990 to 2018, patients with asthma and chronic obstructive pulmonary disease accounted for >45% of the total SCS prescriptions.^15^

Acute SCS usage for exacerbations varies across asthma severities, but is highest in those with the most severe disease.^16^ A systematic literature review conducted in 2020 revealed that 23.2–92.6% of patients with severe or difficult-to-treat asthma were prescribed acute SCS for the treatment of an exacerbation.^16^ Long-term SCS use was reported to range from 1.2 to 30.9% in patients with any degree of asthma severity, and from 20 to 60% in patients with severe or uncontrolled asthma worldwide.^16^ Furthermore, recent population-based studies demonstrate that many people receiving SCS are exposed to relatively high doses over a prolonged period of time, increasing their risk of AEs.^17^, ^18^, ^19^ During a study in the European Union from July 2011 to February 2018 (N = 702,685), 14–44% of the patients with asthma were receiving OCS and 6–9% were "high-OCS users" with an average dose of 5.5–7.5 mg/day for ≥2 years.^18^ Similarly, a study from January 2012 to December 2017 in the United States (N = 435,675) reported that 65% of patients with asthma were receiving OCS over the 2-year follow up, and 19% were classed as "high-OCS users" with an average dose of 5.1–7.1 mg/day over 3 years.^19^ Additionally, a US study of 640,936 individuals with severe asthma demonstrated that 22.5% filled ≥2 and 12.7% filled ≥3 SCS prescriptions annually.^20^ Variation in the prevalence of SCS use globally and regionally may be attributed to multiple factors, including patient age, sex, disease severity, socioeconomic status, level of ICS use, and access to and reimbursement of alternative therapies.^3^^,^^16^^,^^17^^,^^21^ Additional challenges are faced by children with asthma receiving SCS, who are at an increased risk of AEs compared with children with asthma not receiving SCS, yet the prevalence of SCS use in children with asthma remains high.^22^

### Routes of administration and cumulative CS exposure

**Fig. 1:**
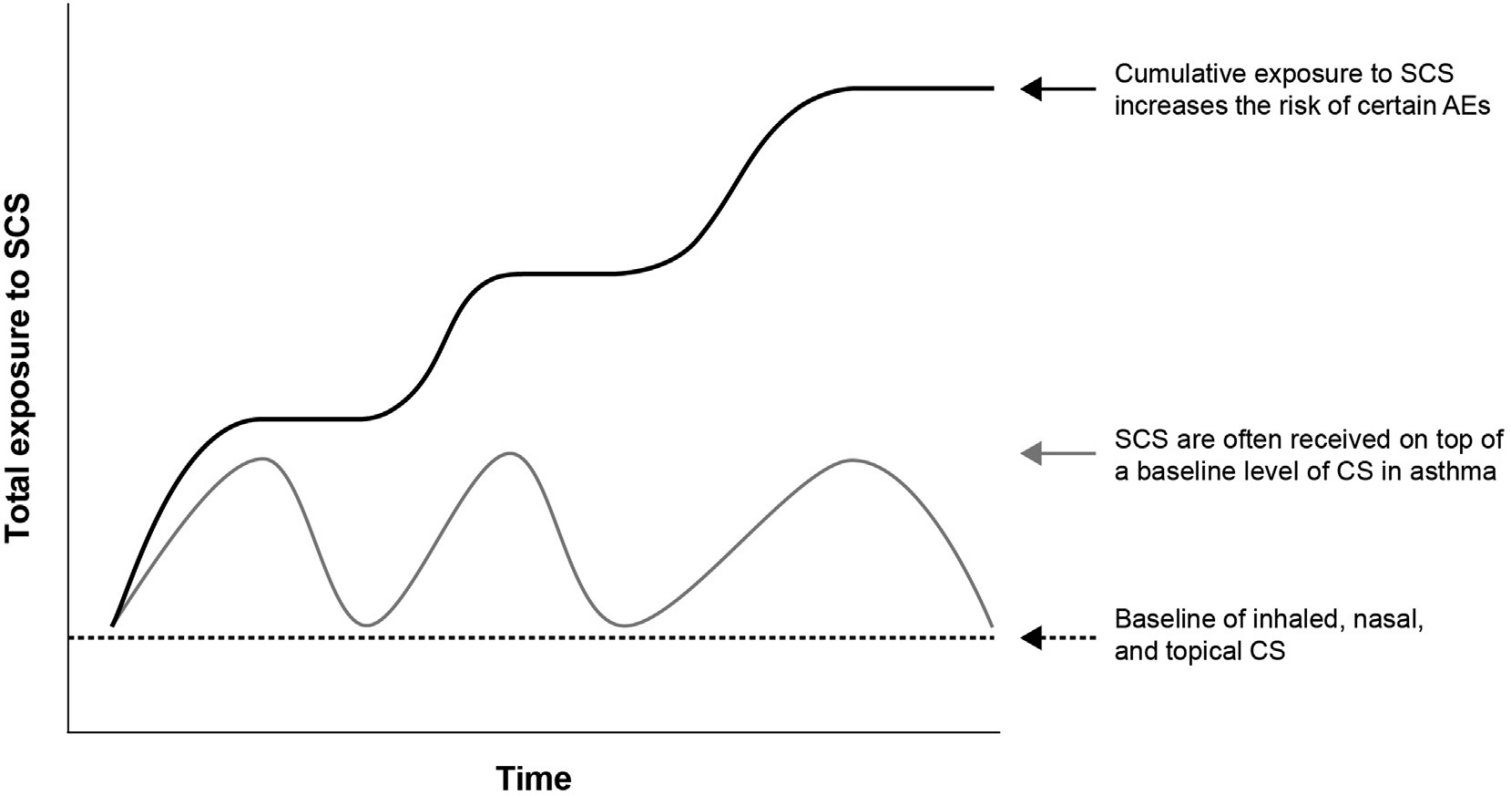
Cumulative exposure to SCS and the associated AEs. AE, adverse effect; CS, corticosteroid(s); SCS, systemic corticosteroid(s).

Overall cumulative CS burden (see Supplemental Appendix 1) is particularly high in patients with asthma, as SCS may be prescribed on top of a background of other potential chronic low-level CS administered through a variety of administration routes and dosing schedules, eg inhaled, nasal, and topical CS (Fig. 1). For example, in an Australian study (N = 124,011), one-quarter of patients with asthma receiving ICS had also been dispensed potentially harmful amounts of cumulative OCS.^23^

SCS are typically administered orally, but are sometimes given as an intramuscular injection.^24^ There is insufficient evidence on whether the acute use of injectable CS is more effective at reducing relapse in patients with asthma attending the emergency department than OCS, potentially owing to factors such as adherence and patient preference.^24^ In addition, it has been reported that 80% of patients with severe asthma do not exhibit an improvement in FEV_1_ when receiving intramuscular CS.^25^ The long pharmacological half-life of injectable CS and better adherence than OCS (owing to the administration method) result in therapeutic efficacy but also a high rate of SCS-related AEs;^26^ thus, injectable CS are another form of SCS that should not be used on a long-term basis.

ICS are recommended for maintenance therapy across all asthma severities, owing to their efficacy in reducing inflammation, improving symptoms, and preventing exacerbations, as well as their improved safety profile compared with SCS owing to reduced systemic absorption.^1^^,^^13^^,^^27^ Nevertheless, some evidence suggests that the risk of AEs with high-dose ICS (see Supplemental Appendix 1) could be similar to that observed with low-dose OCS (Fig. 1),^13^^,^^28^ while high-dose ICS in combination with other CS may further increase the risk of AEs, such as adrenal suppression.^29^^,^^30^ Although these observations need to be further investigated, the potential side effects from high-dose ICS suggest the need to consider these as part of SCS Stewardship, especially when associated with other topical routes of administration.^13^

Type 2 (T2) comorbidities (such as eczema/atopic dermatitis and food allergy) are common in patients with asthma, which adds to the complexity of management and treatment because of the increased risk of exacerbations, poorer asthma control, and polypharmacy.^31^, ^32^, ^33^, ^34^ Analyses of Severe Asthma Network in Italy (SANI) registry data reported that a large proportion of patients with severe asthma also had T2-related comorbidities, such as rhinitis (68.2%) and/or chronic rhinitis with nasal polyps (42.6%), which can occur in combination.^31^ Use of additional steroids for treatment of comorbidities that are administered using alternative methods, such as topical steroids for sinonasal disease or intranasal steroids for nasal polyps,^35^^,^^36^ should also be taken into account when considering total steroid load as, despite their limited absorption, these may lead to a further increased risk of AEs^32^^,^^35^ and healthcare costs.^37^

Furthermore, asthma symptoms may lead to inactivity^1^ that can increase the risk of conditions such as obesity, type 2 diabetes and osteoporosis,^38^ which can be further aggravated by SCS use.^32^^,^^39^

## Burden of SCS-related AEs

### AEs associated with long-term SCS use

It is well documented that SCS-related AEs are highly prevalent in people with severe asthma following long-term use (see Supplemental Appendix 1).^16^^,^^40^^,^^41^ The most common SCS-related AEs (see Supplemental Appendix 2) include osteoporosis, cardiovascular disease, and metabolic complications.^6^^,^^16^^,^^40^^,^^41^ Metabolic-related complications including type 2 diabetes and weight gain (hazard ratio [HR] 1.26 and 1.14, respectively) are associated with a dose-dependent increase in SCS compared with no SCS use (from cumulative exposures [calculated as cumulative prednisolone-equivalent exposure divided by follow-up duration] of 0.5–1 g to ≥10 g compared with >0–<0.5 g).^6^ An increased risk of osteoporosis and fractures in patients receiving SCS is also dose dependent compared with no SCS use.^6^ In 1 study, the adjusted risk in the SCS arm (compared with the non-SCS arm) ranged from 1.14 times greater for weight gain to 3.11 times greater for new osteoporosis/fracture.^6^ The association between SCS use and cardiovascular complications is also well documented.^6^ For example, in a prospective study of two matched cohorts, use of OCS was associated with greatly enhanced risk of coronary heart disease (HR 2.59) and heart failure (HR 3.48).^42^

### AEs associated with acute SCS use

The impact of frequent acute SCS prescriptions, and therefore high levels of cumulative SCS exposure over time, is often underestimated by patients and HCPs.^43^ Importantly, a recent long-term observational study (N = 24,117) comparing patients receiving ≥2 SCS prescriptions with those receiving no SCS (median follow-up time was 7.4 years in the SCS group and 6.4 years in the non-SCS group) demonstrated that serious SCS-related AEs emerge from cumulative threshold doses of 0.5–1 g, which are equivalent to 2–4 lifetime acute CS courses.^6^^,^^43^ This suggests that even short-term SCS use is associated with an increased risk of acute and chronic AEs.^43^ Data from a retrospective cohort study indicate that the number of OCS prescriptions dispensed within a year is strongly associated with AEs, irrespective of dose and duration.^44^ Patients receiving an annual cumulative dose of ≥4 OCS prescriptions (n = 72,063) had 1.29 times the odds of experiencing a new CS-related AE within the year compared with those receiving no OCS (n = 156,373).^44^ The risk of experiencing an AE has been found to rise with increasing OCS use regardless of asthma severity.^45^

In addition to the cumulative effects of frequent SCS courses over time, 2 recent large studies in Taiwan and the United States conducted over 3 years (N = 2,623,327 and 1,548,945, respectively) indicated that the risk of acute and severe AEs including gastrointestinal bleeding, sepsis, venous thromboembolism, fracture, and heart failure was increased in patients who received ≥1 SCS prescription.^46^^,^^47^ However, few data are available regarding the contribution of acute SCS use administered on a background of long-term SCS. A potential future area of research could identify if reducing either the long-term or acute SCS will reduce the risk of future AEs.

### Association between SCS and mortality

Three recent studies indicate that long-term SCS use is associated with a higher risk of mortality compared with no SCS use.^48^^,^^49^ In a nationwide study in Sweden (N = 217,993), long-term OCS use was associated with an increased risk of all-cause mortality (adjusted HR 1.34; 95% confidence interval [CI] 1.24, 1.45; P < 0.001) compared with periodic and non-use.^48^ This observation persisted after adjustment for age and sex.^48^ Similar results were also observed in a Korean population-based study of 466,941 patients (8334 receiving SCS long term), in which the HR of mortality associated with long-term SCS use compared with no SCS use in asthma was 2.17 (95% CI 2.04, 2.31).^49^ Associated mortality rates were dose dependent and were 2.56 and 1.84 times higher in patients receiving high- (≥5.5 mg/day) and low-dose SCS (<5.5 mg/day), respectively, than in patients not receiving SCS.^49^ Results remained significant after adjustment for a number of comorbidities (HR 2.10; 95% CI 1.97, 2.23).^49^ Finally, a recent UK mortality analysis evaluating the association between SCS exposure and mortality found that after adjusting for potential confounders, greater cumulative and average daily SCS exposure was associated with increased mortality.^50^ Patients exposed to a cumulative dose ≥10 g of SCS were more than twice as likely to die as those with cumulative doses of <0.5 g, and patients exposed to an average of ≥7.5 mg/day were ∼4.6 times more likely to die as those exposed to <0.5 mg/day.^50^

The limitations of these studies must be considered. For example, as these studies were each completed in a single country (Sweden, Korea, and the United Kingdom), the results are not likely to take into account variation in clinical characteristics based on country or ethnicity, and the methodologies used did not adjust for all potential confounders.^48^, ^49^, ^50^ In addition, OCS use was based only on collected prescription information, which may not fully reflect patients" actual medication use, and it is unclear whether the higher associated mortality was due to SCS exposure or worsening disease.^48^, ^49^, ^50^ Nonetheless, these studies clearly suggest that there is a potential association between SCS use and mortality and support the need for SCS-sparing strategies in asthma.

### Patient perspective

**Fig. 2:**
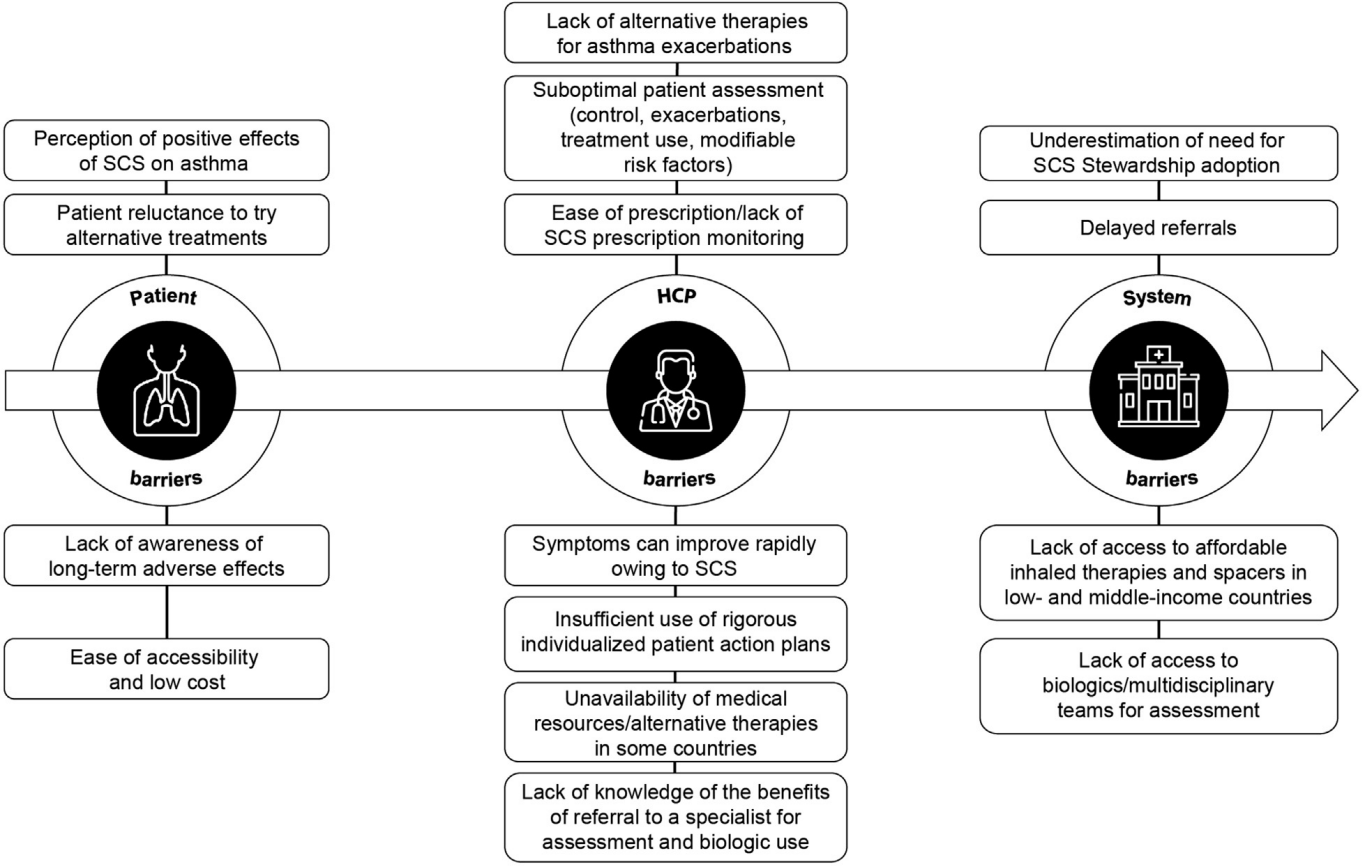
Barriers to SCS Stewardship in asthma. HCP, healthcare provider; SCS, systemic corticosteroid(s).

Although patients may rely on SCS and prefer to use them because of the impacts they have on perceived asthma control and well-being (reviewed in Fig. 2), patient perspectives emphasize the detrimental impact of SCS-related AEs such as weight gain, sleep disturbances, gastric and skin changes, and psychological effects.^21^^,^^40^ Anxiety and depression were respectively 2 and 3.5 times more likely in patients with OCS-dependent asthma than in patients with severe non–OCS–dependent or mild-moderate asthma.^51^ It is therefore not surprising that SCS are associated with a substantial deleterious impact on patients' health-related quality of life (QOL).^52^ In adjusted analyses, use of ≥4 annual SCS prescriptions (N = 624) was associated with a significant reduction in measures of health-related QOL compared with no SCS exposure.^52^ Despite the clear impact on patients' QOL, the true burden of SCS use is relatively unknown as it is not commonly assessed in outcome studies.^53^ It is of great importance for patients and HCPs to be educated on the associated risks and have open dialogues about their medications and AEs.

### Economic and societal burden

There is a substantial societal impact in terms of healthcare resource utilization (HCRU) and costs due to inappropriate SCS exposure,^7^^,^^16^^,^^54^ with the annual cost of SCS-related comorbidities increasing with asthma severity.^5^ In a UK study (9413 matched pairs), HCRU and costs increased annually for patients receiving SCS compared with patients not receiving SCS.^7^ The increase in all-cause AE-associated HCRU and costs was dose dependent, with SCS exposure of ≥7.5 mg/day resulting in 2.3–3.0 times greater HCRU from AEs compared with no exposure to SCS.^7^ Similar findings were also reported in a US matched-cohort study of patients with persistent asthma, in which total healthcare costs and long-term complication-related costs were significantly greater in patients who had ≥4 SCS claims ($22,311 and $2,647, respectively) than in those not receiving SCS.^54^

Furthermore, an analysis of data from the SANI registry estimated the total annual healthcare cost of OCS-related AEs for patients with severe asthma in Italy at €242.7 million, representing an incremental expenditure of ∼€110.6 million and ∼€75.2 million compared with the non-asthmatic and moderate asthma populations, respectively.^5^ Indirect costs, eg work productivity losses due to SCS-related AEs, should also be considered.^55^ A US questionnaire (N = 1109) demonstrated that the mean work impairment (= mean hours of work missed + hours worked × impairment while working due to asthma) of employed patients with severe asthma receiving long-term maintenance SCS was 34%, which decreased to 17% in patients receiving biologics, suggesting that there is an economic reasoning for reducing inappropriate SCS use and prescribing biologics to eligible patients.^55^

### Implementing SCS-sparing strategies and SCS Stewardship

Accumulating evidence indicates that SCS Stewardship, defined as a collaborative systematic effort to protect patients and reduce the harm from inappropriate or cumulative SCS use, is urgently needed.^12^^,^^56^ This can be achieved through a multidisciplinary effort to 1) adopt SCS-sparing strategies, ie, "preventative" measures to improve asthma control and avoid/minimize exacerbations and the subsequent need for SCS use, and 2) implement SCS Stewardship through recognition of the potential benefits of SCS to patients, while also providing a structured approach to carefully prevent inappropriate use (eg, tapering, monitoring of AEs).^12^^,^^21^ Patient-, HCP- (including pharmacists), and system-related barriers to adoption of SCS Stewardship, reviewed in Fig. 2, must be addressed to reduce inappropriate SCS use and minimize its burden on the patient and society.^21^^,^^40^^,^^57^

### SCS-sparing strategies

**Fig. 3:**
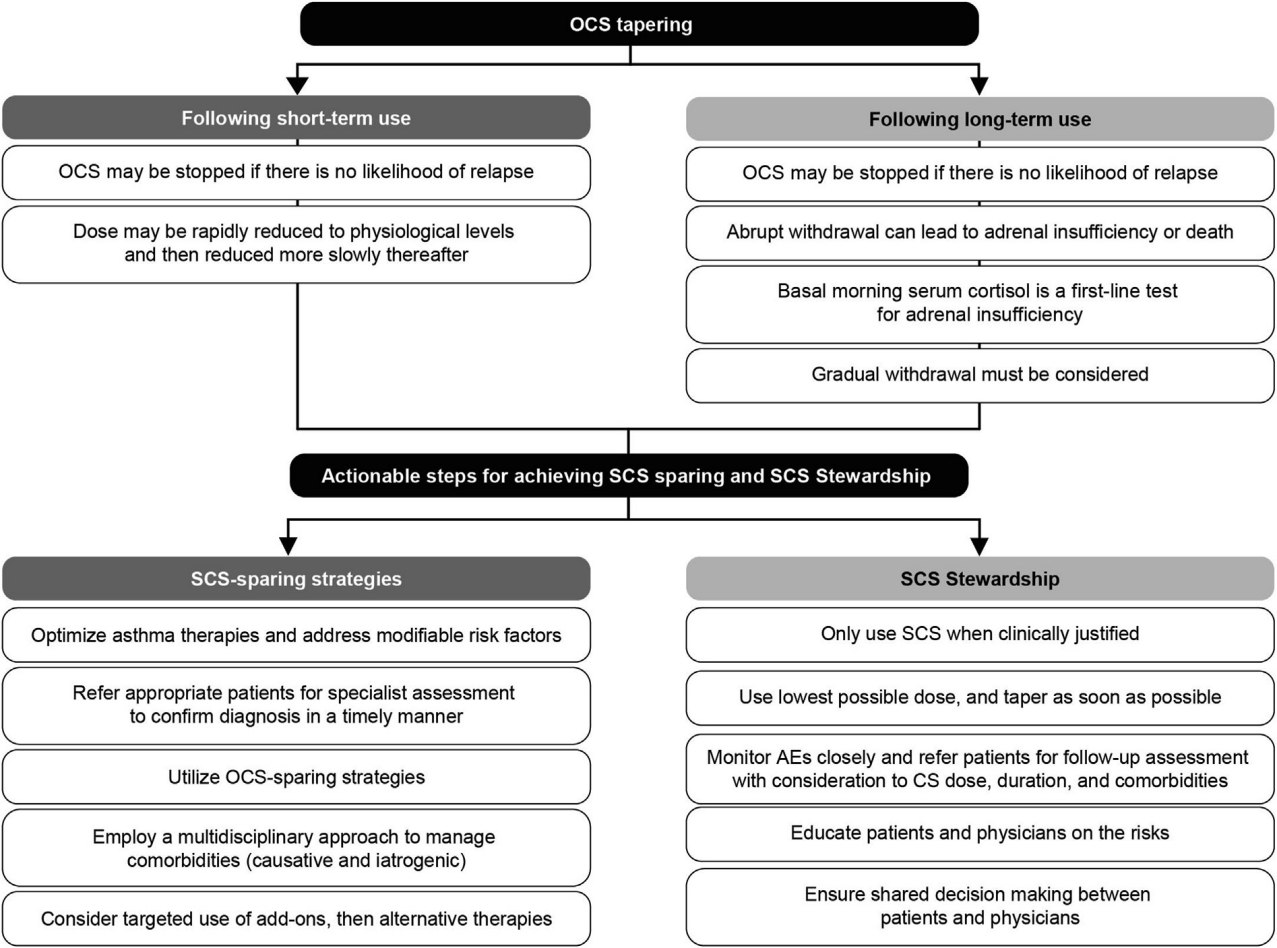
Considerations for OCS tapering and actionable steps to achieve SCS sparing and SCS Stewardship. AE, adverse effect; CS, corticosteroid(s); OCS, oral corticosteroid(s); SCS, systemic corticosteroid(s).

Strategies for SCS tapering and actionable steps for achieving SCS sparing and Stewardship are reviewed in Fig. 3.

### Optimize maintenance asthma therapies and address modifiable risk factors

Primary prevention of exacerbations requiring SCS by optimizing maintenance asthma therapies, in line with guideline-recommended care, is the first crucial step in reducing inappropriate SCS use.^1^ Real-world evidence from a recent US database study suggests that OCS are often prescribed instead of stepping up maintenance therapy; ∼25% of "high-OCS users" were prescribed short-acting β_2_-agonists only during the 6-month baseline study period.^19^ Patients with milder severities of asthma can also receive inappropriate doses of SCS, which may be prevented with earlier assessment and optimization of therapies.^58^ Modifiable risk factors should be assessed as they may contribute to poor asthma control (Table 1).^1^ For instance, inadequate adherence to ICS and/or incorrect inhaler technique may lead to exacerbations, requiring avoidable SCS to be prescribed.^23^ If a patient's asthma remains uncontrolled after modifiable risk factors are addressed (Table 1), then specialist referral is required.^1^

**Table 1:** Alternative therapies and modifiable risk factors.

Alternative therapies to SCS	Modifiable risk factors
• Add-on therapies (if not already attempted) ‒ LTRA ‒ Tiotropium/triple therapy ‒ Azithromycin ‒ Biologics • Sublingual immunotherapy	• Smoking • Comorbidities • Incorrect diagnosis • Major psychological problems • Major socioeconomic problems • Food allergy • Allergen exposure if sensitized^a^ and inhaled pollutants • Incorrect inhaler technique • Suboptimal treatment adherence • Medication-related AEs • Regular use or overuse of SABA

AE, adverse effect; LTRA, leukotriene receptor agonist; SABA, short-acting β_2_-agonist(s); SCS, systemic corticosteroid(s).

a Treatment includes allergen-specific immunotherapy, where appropriate.

### Timely diagnosis and referral for systematic assessment by a multidisciplinary team

Patients with uncontrolled asthma need to be identified and systematically assessed to aid differentiation of difficult-to-treat and treatment-resistant asthma from severe asthma,^1^^,^^59^ in order to identify those who may benefit from referral to specialist care for further endotypic evaluation (to identify the mechanistic cause), treatment optimization, or innovative targeted therapies.^1^^,^^2^^,^^12^^,^^60^ In addition, distinguishing symptoms that can mimic asthma, such as vocal cord dysfunction and dysfunctional breathing, is important in ensuring that an accurate diagnosis and appropriate treatment are received for conditions that do not require SCS.^34^^,^^61^ Systematic assessment by a dedicated multidisciplinary team at a severe asthma center can provide comprehensive evaluation to optimize treatment of asthma and related comorbidities and outcomes for patients.^62^ Experts advocate that greater collaboration between specialist and primary care (eg, primary care physicians [PCPs], pulmonologists, allergists, and pharmacists) is important to ensure timely referral.^36^ Studies indicate that systematic assessment of patients at dedicated severe asthma centers is associated with improved QOL and asthma control, reduced healthcare use, and reduced burden to the patient.^63^^,^^64^

Ideally, all patients with severe or difficult-to-treat asthma should be referred to specialist care whenever possible; however, many patients with asthma who could benefit from such a referral remain in primary care, which may lead to further inappropriate SCS use.^65^ Delayed referrals may occur as a result of a lack of coordination within the healthcare system or restrictive referral criteria (eg, only those who meet specific severity criteria will qualify).^65^ Timely and appropriate referral of patients with asthma to specialist care provides access to add-on therapies (including conventional therapies and biologics; Table 1), decreases long-term SCS use, improves asthma control, and may reduce morbidity and mortality.^63^ However, access to treatment options like biologics is limited in some areas globally, especially in lower-income countries,^57^ and a recent review of severe asthma biologic prescription criteria across 28 countries revealed that there is substantial variation in criteria for use, including factors such as a patient requirement for long-term OCS use.^66^ The literature supports >2 courses of OCS in the previous year or a cumulative SCS dose of 0.5–1 g/year (equivalent to 2–4 courses/year) as a trigger for referral of a patient for specialist evaluation and care because of the increased risk of AEs.^6^^,^^67^ However, of the 8% of patients with severe asthma in a UK database study (16,409 of 207,557), 72% had not been reviewed or referred to a specialist in the last year and 56% had no record of ever being referred.^65^ In countries such as Belgium, referral alerts are embedded in primary care software to allow PCPs to identify patients who meet certain SCS-related criteria (eg, medium-to-high-dose ICS-LABA with ≥1 OCS prescription) and should thus be referred for specialist assessment.^36^ Such alerts should incorporate pharmacy data with patient files to ensure that complete use of SCS is captured.^36^ Integration of referral alerts into primary care and pharmacy software may improve the communication between PCPs and specialist care and accelerate identification of patients who may benefit from specialist referral.^36^^,^^67^ While global recommendations suggest that some clinical situations warrant referral to specialist care for assessment/treatment, referral is not always possible owing to the variation in asthma care, policy, and health systems in different countries.^1^

### Assess biomarkers and implement a personalized treatment approach

Severe asthma comprises multiple phenotypes that underlie the need for a personalized treatment approach through specialist care.^68^ A personalized approach to asthma management ensures that treatment is tailored to suit individual patient needs.^68^^,^^69^ Global recommendations for asthma management suggest that treatment decisions should take into account patients" disease characteristics and phenotypes to predict their response to treatment.^1^ A coordinated effort to improve phenotyping and biomarker-driven approaches to severe asthma therapy is needed.^2^

Although there may be some overlap, T2 biomarkers (fractional exhaled nitric oxide [FeNO], blood eosinophils, and IL-6) play a central role in successful SCS Stewardship in asthma as they can aid in determining treatment responsiveness to SCS and SCS-sparing add-on therapies.^1^^,^^69^^,^^70^ In patients with T2 inflammation (despite high-dose ICS), SCS use may be efficacious and clinically justified as they primarily suppress T2 inflammation,^71^ whereas in patients with non-T2 endotypes, escalating doses of SCS can contribute to the SCS burden with little/no therapeutic effect.^1^^,^^16^ If other treatment options are not viable, the Global Initiative for Asthma (GINA) report 2022 suggests that biomarkers for T2 inflammation (FeNO, blood eosinophils, and sputum [if available in the clinical setting]) are assessed prior to OCS treatment as OCS can suppress these biomarkers.^1^ For example, blood eosinophils are used as a marker in risk prediction tools for exacerbations and have also been linked to a response to biologics.^72^^,^^73^ Furthermore, evolving advances in our understanding of the microbiome suggest that it may have a role as a biomarker for T2 asthma and in the future this may lead to the identification of new biomarkers to facilitate treatment choices.^70^ Thus, the initial assessment of biomarkers for T2 inflammation prior to SCS initiation (or at the lowest possible dose)^1^ may determine the likelihood of clinical response,^74^ and help reduce additional unnecessary exposure.^1^ In addition, 50.3–83.8% of patients with severe asthma are most likely to have an eosinophilic phenotype, which if identified (eg, through an evidence-based eosinophil algorithm that utilizes variables accessible to both primary and secondary care) may help to reduce severity and HCRU owing to earlier introduction of appropriate therapies.^75^^,^^76^ Easily measured, available biomarkers should be assessed early in patient care, and strategies to guide treatment plans based on patient response to therapies should be part of primary care interventions and included in specialist software.^1^^,^^16^^,^^73^^,^^77^

### Utilize SCS-sparing therapies

SCS-sparing therapies can be utilized to eliminate inappropriate SCS use.^36^^,^^78^ In patients with uncontrolled asthma despite high-dose ICS/LABA and no evidence of T2 inflammation, additional non-biologic add-on treatments should be considered by a specialist, eg additional controller therapy with a long-acting muscarinic antagonist (LAMA; triple therapy) and low-dose azithromycin (adults).^1^ For example, tiotropium as an add-on to ICS and LABA has been shown to reduce the risk of asthma exacerbations and provide modest sustained bronchodilation,^79^^,^^80^ and triple therapies (LAMA, ICS, and LABA) have been shown to improve lung function.^81^

In patients with T2 inflammation, add-on biologic T2-targeted therapies should be considered prior to the use of long-term SCS (Table 1).^1^ As well as reducing exacerbations, therapy with several targeted biologics, such as with omalizumab,^82^ mepolizumab,^83^ benralizumab,^84^ and dupilumab,^85^ has been shown to reduce or eliminate SCS use in severe asthma, emphasizing the importance of considering add-on therapies to minimize SCS use.^16^^,^^83^, ^84^, ^85^ In addition, some patients with asthma develop airflow limitation due to airway remodeling,^1^ which may be preventable with earlier administration of alternative treatments, thus preserving lung function. This is demonstrated by improvement in lung function while maintaining acute bronchodilator responsiveness with targeted biologic therapies.^85^^,^^86^

According to national and international registry data, OCS sparing should be considered as the primary outcome in managing severe asthma owing to the impact that OCS exposure has on the patient's QOL (eg absence from work or decreased productivity) and reduction in AE-associated costs.^55^^,^^78^

## SCS Stewardship

### Only use SCS when clinically justified

A treatment aim is to achieve remission. A proposed criterion for asthma remission is no need for SCS;^87^ therefore, prevention of inappropriate short- and long-term SCS use is important, where possible.^88^ In a Delphi consensus relating to OCS use and tapering, asthma experts concluded that long-term OCS use is not appropriate when other treatments are available; however, if no alternative treatments are suitable, the lowest steroid dose should be utilized (≤5 mg/day).^89^ GINA suggests that OCS are effective treatments for acute exacerbations^1^ as their early use has been shown to reduce hospitalizations and relapses to additional care.^1^^,^^90^^,^^91^ However, consideration has to be made to the emerging evidence describing the risks associated with repeated acute courses of OCS,^6^^,^^44^^,^^47^^,^^89^ which has recently been acknowledged in the GINA 2021 update slide deck^10^ and Italian asthma recommendations.^11^ Unfortunately, there is a lack of alternative options for targeted treatment of acute exacerbations, but these may be reduced through frequent monitoring of symptom control and risk factors, optimization of standardized treatments to prevent exacerbations, and targeted biologics to reduce exacerbations and OCS use.^1^^,^^78^^,^^89^ Global recommendations suggest that monitoring of symptom control should occur regularly, using tools such as asthma questionnaires, not just when patients present with worsening asthma control.^1^ It is of utmost importance to assess the patient's asthma after an exacerbation in a timely manner to identify the cause and triggering factors (eg, influenza), and to regularly monitor asthma control to guide necessary changes in the patient's treatment (eg, smoking cessation and improved adherence to treatments), thus reducing the likelihood of further exacerbations.^1^^,^^92^ Asthma action plans should be provided and reviewed with patients regularly to reduce the risk of further exacerbations and ensure that inappropriate SCS use is not incorporated into a patient's treatment.^1^^,^^93^

### When using SCS long term, use the lowest possible dose, and taper as soon as possible

Tapering involves slowly reducing the dosage of a medicine to the lowest possible dose, often followed by continuous monitoring.^1^^,^^94^ Despite experts agreeing that OCS tapering is important to avoid burden to the patient, a standardized approach is lacking.^89^ OCS tapering regimens using biologics such as mepolizumab^83^ have been proposed in some trials. The PONENTE trial (the largest OCS-sparing trial to date in severe asthma) investigated the safety and effectiveness of a rapid, individualized, steroid-reduction algorithm, including adrenal insufficiency (AI) monitoring, after benralizumab initiation.^95^ The primary endpoints of the trial included whether patients achieve 100% reduction in daily OCS use and/or achieve OCS dosage of ≤5 mg/day if AI prevented further reduction (both sustained over ≥4 weeks without worsening of asthma).^95^ The trial used an algorithm to taper OCS when prednisolone dosage is ≤5 mg/day, and included a longer maintenance phase (∼24–32 weeks) to allow assessment of asthma control following completion of tapering.^95^ In the trial, 81.94% of adults with asthma receiving prednisolone for ≥3 months (n = 598) eliminated OCS use, or their daily use was reduced to ≤5 mg/day if AI prevented further reduction, with benralizumab.^29^ In a Delphi consensus on OCS tapering, asthma experts reached consensus on an OCS tapering algorithm.^89^ Tapering should be attempted in all patients with asthma receiving OCS long term, especially if other treatments are available (Fig. 3).^89^ Tapering should be personalized to each patient as certain factors will influence the speed and rhythm of tapering to CS cessation, or reaching the lowest possible dose during long-term therapy.^89^ Standardized tapering schemes (eg, from the PONENTE trial) should be utilized and individualized based on the patient and be made available for use internationally.

### Identify and manage adrenal insufficiency

One challenge that can impede successful tapering of SCS is that withdrawal of long-term CS can result in suppression of the hypothalamic-pituitary-adrenal (HPA) axis, leading to AI, which becomes evident after SCS withdrawal.^95^^,^^96^ AI cannot be accurately predicted by CS dose or duration of treatment.^94^ Patients receiving OCS are at greatest risk of AI compared with those receiving nasal and topical CS, and ICS, but consideration should be given to their cumulative effect.^96^ As AI can have serious consequences for the patient, tapering of OCS must be done carefully and slowly (Fig. 3),^21^^,^^94^^,^^96^ and with guidance from an endocrinologist, when appropriate and feasible.^56^ However, the risk of AI becomes more apparent at doses of OCS ≤7.5 mg, which correspond to physiological levels.^29^ The PONENTE trial suggests an OCS tapering protocol that includes an HPA axis integrity evaluation to identify AI after 4 weeks in patients who have reduced their OCS dosages to 5 mg/day, or 4 weeks after the first dose of benralizumab before OCS reduction in those with 5 mg/day OCS at baseline.^95^ Adrenocorticotropic hormone stimulation tests are also suggested within 1 week after the HPA evaluation if partial AI has been found.^95^

### Monitor use and AEs closely

Although SCS use has not historically been closely monitored in the past, geographic mapping of severe uncontrolled asthma across the USA has been recently completed in order to assess CS usage to guide therapies and improve outcomes in patients with severe asthma.^20^ After adjusting for variables such as cities with the greatest disease burden, some counties (smaller regions within states) were found to have higher rates of SCS prescriptions and rates of asthma-related mortality than others, highlighting the need for monitoring of SCS prescriptions so that SCS Stewardship strategies and education can be implemented in the areas with the highest need.^20^

It was concluded in a Delphi consensus that when SCS use is deemed appropriate, patients should be closely monitored for glycemic control, bone density, and blood pressure.^89^ Techniques such as a glucocorticoid toxicity index are being developed to facilitate monitoring of patients while tapering.^97^ Identification of use of recurrent courses of SCS is important; this can be achieved through alerts at the pharmacy or primary care level and should be built into primary care software as a safeguard.^21^

### Shared decision making between patients and healthcare providers

The Patient Charter for severe asthma was created to empower patients through mobilizing stakeholders in asthma healthcare to address the unmet need and burden in severe asthma.^60^ The charter consists of 6 core principles, one of which states: "I deserve not to be reliant on oral corticosteroids",^60^ highlighting the need for patients to be empowered to discuss alternative treatment options with their HCP. The OCS Stewardship statement (2018), endorsed by multiple asthma organizations, highlights the need to educate patients about the risks associated with OCS and available treatment options.^12^ More than half of patients surveyed by the Asthma and Allergy Foundation of America were not aware of other innovative treatment options for their severe asthma.^12^

Shared decision making between patients and HCPs should be encouraged to ensure that care focuses on reducing the impact of AEs and symptoms on patients" daily lives.^60^ Risk prediction formulas have been developed that estimate the risk of an AE (eg, osteoporosis) occurring after OCS exposure; this may help patients to understand their individualized level of risk and guide shared decision making between patients and HCPs.^98^ Shared decision making has been strongly supported by asthma experts, especially during the SCS tapering process,^89^ and has also been shown to benefit other aspects of care, such as adherence to medication and health-related QOL.^99^

## Conclusions

It is of utmost importance for HCPs to take ownership of increasing awareness of the risks associated with inappropriate long- and short-term SCS use by implementing SCS Stewardship and by following the strategies outlined in this review to prevent and reduce the harm to patients. SCS use should be reduced to the lowest possible dose and managed through tapering and sparing strategies. Close monitoring of SCS use is crucial, as well as frequent reassessment of treatments, asthma control, exacerbation occurrence, and modifiable risk factors. Although the focus remains on OCS, other CS (inhaled, topical, and injectable CS) contribute to systemic steroid load and should be considered as part of SCS Stewardship.

Future directions include additional research to develop alternative treatments to SCS for potentially life-threatening exacerbations and development of standardized guidelines to aid HCPs in adopting efficient tapering schemas that are individualized for each patient and the severity of their asthma. Increasing access and affordability of asthma therapies, including SCS alternatives, is of great importance. There is also a need for global and local guideline reform to ensure adoption of structured SCS Stewardship programs in clinical practice to enable a robust, joined-up approach.

## Abbreviations

AE; adverse effect, AI; adrenal insufficiency, CI; confidence interval, CS; corticosteroid(s), FeNO; fractional exhaled nitric oxide, FEV_1_; forced expiratory volume in 1 second, GINA; Global Initiative for Asthma, HCP; healthcare provider, HCRU; healthcare resource utilization, HPA; hypothalamic-pituitary-adrenal, HR; hazard ratio, ICS; inhaled corticosteroid(s), LABA; long-acting β_2_-agonist(s), LAMA; long-acting muscarinic antagonist, OCS; oral corticosteroid(s), PCP; primary care physician, QOL; quality of life, SABA; short-acting β_2_-agonist(s), SANI; Severe Asthma Network in Italy, SCS; systemic corticosteroid(s), T2; type 2.

## Authors" contributions

All authors contributed equally to the conception, design, acquisition of resources and development of the paper. All authors approved the final version.

## Ethics approval and consent to participate

Not applicable.

## Consent for publication

The authors consent to the publication of this work in World Allergy Organization Journal.

## Funding

This manuscript was funded by a grant from AstraZeneca. The authors retained full control. AstraZeneca provided a review for scientific accuracy and did not participate in the content development.

## Availability of data and material

Not applicable.

## Declaration of competing interest

MA-A, LB, CM, AY declare that they have no competing interests.

EB reports that he has undertaken clinical trials through his employer, Wake Forest School of Medicine and the University of Arizona for AstraZeneca, MedImmune, Boehringer Ingelheim, Genentech, Novartis, Regeneron, and Sanofi Genzyme; personal fees as a paid consultant for ALK-Abello, AstraZeneca, GlaxoSmithKline, MedImmune, Novartis, Regeneron, Sanofi Genzyme, and TEVA, outside the submitted work.

GWC reports having received research grants as well as being a lecturer or having received advisory board fees from A. Menarini, Alk-Abello, Allergy Therapeutics, AstraZeneca, Chiesi Farmaceutici, Firma, Genentech, Guidotti-Malesci, GlaxoSmithKline, Hal Allergy, Mylan, Novartis, Regeneron, Roche, Sanofi-Aventis, Sanofi-Genzyme, Stallergenes-Greer, Valeas, Om-Pharma, outside of the submitted work.

AK non-financial support from AstraZeneca; personal fees from AstraZeneca, Behring, Boehringer Ingelheim, Covis, Cipla, GSK, Eisai, Novartis, NovoNordisk, Pfizer, Sanofi, Teva, Trudel, Valeo, outside the submitted work.

NP reports personal fees from Novartis and Nutricia, outside the submitted work.

NR reports grants and personal fees from Boehringer Ingelheim, Novartis, Pfizer, GSK and personal fees from MSD, AstraZeneca, Chiesi, Sanofi, Zambon, outside the submitted work.

DR reports personal fees from Viatris, Regeneron, and Novartis, outside the submitted work.

YT reports support from AstraZeneca, Kyorin Pharmaceuticals, and Sanofi, outside the submitted work.

DP reports advisory board membership with Amgen, AstraZeneca, Boehringer Ingelheim, Chiesi, Circassia, Viatris, Mundipharma, Novartis, Regeneron Pharmaceuticals, Sanofi Genzyme, Teva Pharmaceuticals and Thermofisher; consultancy agreements with Amgen, AstraZeneca, Boehringer Ingelheim, Chiesi, GlaxoSmithKline, Viatris, Mundipharma, Novartis, Pfizer, Teva Pharmaceuticals and Theravance; grants and unrestricted funding for investigator-initiated studies (conducted through Observational and Pragmatic Research Institute Pte Ltd) from AstraZeneca, Boehringer Ingelheim, Chiesi, Circassia, Viatris, Mundipharma, Novartis, Pfizer, Regeneron Pharmaceuticals, Sanofi Genzyme, Teva Pharmaceuticals, Theravance and UK National Health Service; payment for lectures/speaking engagements from AstraZeneca, Boehringer Ingelheim, Chiesi, Cipla, GlaxoSmithKline, Viatris, Mundipharma, Novartis, Pfizer, Regeneron Pharmaceuticals, Sanofi Genzyme and Teva Pharmaceuticals; payment for travel/accommodation/meeting expenses from AstraZeneca, Boehringer Ingelheim, Circassia, Mundipharma, Novartis, Teva Pharmaceuticals and Thermofisher; funding for patient enrolment or completion of research from Novartis; stock/stock options from AKL Research and Development Ltd which produces phytopharmaceuticals; owns 74% of the social enterprise Optimum Patient Care Ltd (Australia and UK) and 74% of Observational and Pragmatic Research Institute Pte Ltd (Singapore); 5% shareholding in Timestamp which develops adherence monitoring technology; is peer reviewer for grant committees of the UK Efficacy and Mechanism Evaluation programme, and Health Technology Assessment; and was an expert witness for GlaxoSmithKline.

## Acknowledgments

This manuscript was endorsed by the World Allergy Organization and the Respiratory Effectiveness Group. Editorial support was funded by 10.13039/100004325AstraZeneca and was provided by Katherine Hardy, PhD of Helios Medical Communications, Oxford, Oxfordshire, UK.

## Submission declaration

The authors declare that this is an original work that has not been previously published in any form. The manuscript is not being considered for publication elsewhere.

## Agreement to publish

All authors have read and approved the submitted manuscript and consent to publish the work.
