# Supplementary material for: Systemic corticosteroids in asthma: A call to action from World Allergy Organization and Respiratory Effectiveness Group
Source: World Allergy Organ J. 2022 Dec 10;15(12):100726. doi: 10.1016/j.waojou.2022.100726 (PMC9761384; doi:10.1016/j.waojou.2022.100726)
Supplement: Multimedia component 1 [file mmc1.pdf]

Supplemental Appendix 1. Summary of key terminology

Term	***Definition***	
Short/acute courses	40–50 mg/day for 3–7 days	
Long-term use	Dosing exceeding 7 days	The GINA report recommends that long-term, low-dose OCS is used as an add-on maintenance therapy in patients with uncontrolled severe asthma (defined as poor control despite optimal therapy with high-dose ICS) with or without LABA and other controllers, but only if contributing factors and side effects are taken into account
Low dose	OCS: typically ≤7.5 mg/day	
*High-dose ICS**	From 200 to >2000 mcg/day depending on which ICS is prescribed in adults	*Total daily high-dose ICS examples according to GINA recommendations:*<br>• *Fluticasone furoate (DPI) 200 mcg/day*<br>• *Beclomethasone dipropionate (pMDI, standard particle, HFA) >1000 mcg/day*

*Medium-dose ICS**	From 100 to 1000 mcg/day depending on which ICS is prescribed in adults	*Total daily medium-dose ICS examples according to GINA recommendations:*<br>• *Fluticasone furoate (DPI) 100 mcg/day*<br>• *Beclomethasone dipropionate (pMDI, standard particle, HFA) >500–1000 mcg/day*
*Low-dose ICS**	From 80 to 5000 mcg/day depending on which ICS is prescribed in adults	*Total daily low-dose ICS examples according to GINA recommendations:*<br>• *Ciclesonide (pMDI, extrafine particle, HFA) 80–160 mcg/day*<br>• *Beclomethasone dipropionate (pMDI, standard particle, HFA) 200–500 mcg/day*
Cumulative use	OCS: ≥450 mg within a 90-day period (corresponding to ≥5 g/day)	
Inappropriate use	Courses of SCS that are not clinically justified	

*Daily ICS doses are shown as metered doses. See product information for delivered doses

DPI, dry powder inhaler; GINA, Global Initiative for Asthma; HFA, hydrofluoroalkane propellant; ICS, inhaled corticosteroid(s); LABA, long-acting β_2-agonist(s); mcg, micrograms; OCS, oral corticosteroid(s); pMDI, pressurized metered dose inhaler; SCS, systemic corticosteroid(s)
