# Supplementary material for: Systemic corticosteroids in asthma: A call to action from World Allergy Organization and Respiratory Effectiveness Group
Source: World Allergy Organ J. 2022 Dec 10;15(12):100726. doi: 10.1016/j.waojou.2022.100726 (PMC9761384; doi:10.1016/j.waojou.2022.100726)
Supplement: Multimedia component 2 [file mmc2.pdf]

Supplemental Appendix 2. SCS-associated AEs

Duration of exposure	***AE***
Acute	**Gastrointestinal**: peptic ulcers
	**Infections**: pneumonia, sepsis, tuberculosis
Chronic	**Metabolic**: diabetes mellitus type 2, obesity, dyslipidemia
	**CV**: CV events, hypertension, hypercholesterolemia
	**Bone related**: osteoporosis, fracture
	**Psychiatric and affective disorders**: anxiety, depression, psychosis, irritability, agitation, insomnia
	**Other**: glaucoma, cataracts, HPA axis suppression, herpes zoster, chronic kidney disease, sleep apnea

AE, adverse effect; CV, cardiovascular; HPA, hypothalamic-pituitary-adrenal; SCS, systemic corticosteroid(s)
